# Preface: special topic on physics of the BESIII experiment

**DOI:** 10.1093/nsr/nwab201

**Published:** 2021-11-12

**Authors:** Yifang Wang

**Affiliations:** High Energy Physics, Chinese Academy of Sciences, China; Guest Editor of Special Topic; Editorial Board Member of NSR

The Beijing electron positron collider (BEPC) was constructed in the 1980s and its upgraded version (BEPCII) with a center-of-mass energy region of 2–5 GeV and a maximum luminosity of 1 × 10^33^ cm^−2^ s^−1^ started operation in 2008. The colliding events are recorded by a newly designed detector, called the Beijing spectrometer (BESIII), based on state-of-the-art technologies. Physicists working at the BESIII experiment formed the BESIII Collaboration in 2005, which is a large international collaboration consisting of more than 500 physicists from 80 institutions in 17 countries. Over the course of more than ten years, about 400 physics papers have been published in internationally renowned journals covering a large variety of physics topics and notable achievements, including the following.

- Discovery of the ‘four-quark matter’ *Z*_*c*_(3900), *Z*_*c*_(4020) and Z_*cs*_(3985), the fine structure of *Y*(4260) and a new production mode for *X*(3872).
- Precision measurements of the standard model (SM) parameters, the τ-lepton mass and |*V*_*cd*_| and |*V*_*cs*_| CKM matrix elements.
- A huge sample of *J*/ψ decays have provided a deeper understanding of the glueballs, the resonant structures at the proton-antiproton mass threshold, as well as possibilities of searching for other light exotic hadrons.
- Precision measurements of low-energy *e*^+^*e*^−^ annihilation cross sections and complex phases in *D*-meson decays provide important input for experiments at other energies, and measurements of the purely leptonic and semileptonic decay rates of *D* and *D*_*s*_ mesons provide sensitive tests of lattice QCD calculations.
- The discovery of the polarization of hyperons produced in *J*/ψ decays provides new and unique opportunities for high-sensitivity searches for non-SM sources of *CP* violation.
- Investigations of time-like electromagnetic form factors of nucleons, Λ, and Λ_*c*_ baryons revealed unexpected threshold behaviors and motivated measurements for other stable baryons.

A collection of review articles in this issue of the journal will cover some of the most important results and future prospects for the next ten years, including charm physics, charmonium and charmonium-like exotic states, light hadron spectroscopy, QCD studies with light meson decays, properties of baryons and new physics searches. These represent the advancement of our understanding on tau-charm physics in the last decades. An interview with Luciano Maiani, who proposed the existence of the charm quark and was the Director General of CERN from 1999 to 2003, is also included in this Special Topic.

BEPCII was originally intended to operate at a variety of energy points in accordance with the physics plan that was articulated in its 2009 yellow book [1]. To date, BESIII has accumulated 10 billion *J*/ψ events, 3 billion ψ(2*S*) events, an integrated luminosity of 3 fb^−1^ at the ψ(3770) resonance and about 25 fb^−1^ at energies above ψ(3770), as was collected in the BESIII physics book in 2020 [2]. An upgrade that would increase the center-of-mass energy from 5 to 5.6 GeV and dramatically increase the luminosity at higher energies was requested by BESIII/BEPCII as part of their proposed program for the next decade [2] and was approved recently by the Chinese Academy of Sciences (CAS). With data from the next three years and more data after the upgrade, BESIII is able to measure charm decays with a three-fold improvement in the precision and to investigate the 4.7–5.6 GeV energy region that has been barely touched previously. The latter opens a window for new exotic hadrons with charm quarks.

BESIII as the world’s premier experiment for physics in the charm and tau energy region is an exemplary, large-scale and ever-expanding international collaboration that is paving the way for Chinese physicists to take a world-leading role in the pursuit of a basic understanding of nature.

We thank the BESIII Collaboration, the BEPCII and the IHEP supporting staff, as well as funding agencies including MOST, NSFC, and CAS in China, and those of foreign countries in the BESIII Collaboration.
